# Changes in tear protein profile in dogs with keratoconjunctivitis sicca following topical treatment using cyclosporine A

**DOI:** 10.14202/vetworld.2021.1711-1717

**Published:** 2021-06-30

**Authors:** Metita Sussadee, Rucksak Rucksaken, Phattara-Orn Havanapan, Onrapak Reamtong, Aree Thayananuphat

**Affiliations:** 1 Department of Veterinary Technology, Faculty of Veterinary Technology, Kasetsart University, Bangkok, Thailand; 2 Institute of Molecular Biosciences, Mahidol University, Salaya campus, Nakhonpathom, Thailand; 3 Department of Molecular Tropical Medicine and Genetics, Faculty of Tropical Medicine, Mahidol University, Bangkok, Thailand; 4 Department of Companion Animal Clinical Sciences, Faculty of Veterinary Medicine, Kasetsart University, Bangkok, Thailand

**Keywords:** cyclosporine, dog, keratoconjunctivitis sicca, proteomic, tear protein

## Abstract

**Background and Aim:**

Keratoconjunctivitis sicca (KCS) is a chronic inflammatory ocular disease that occurs in many dog breeds worldwide. This study aimed to investigate the tear protein pattern of healthy dogs, KCS dogs, and KCS dogs after treatment with cyclosporine A (CsA).

**Materials and Methods:**

Twenty-eight dogs of any breed were enrolled in the study. The subjects were divided into three groups: Healthy, KCS, and CsA-treated dogs. Tear samples were collected using Schirmer strips. Tear proteins extracted from the strips were analyzed using two-dimensional electrophoresis. For the first dimension, total protein from tears was separated by isoelectric focusing. The second dimension was performed using 12.5% sodium dodecyl sulfate-polyacrylamide gel electrophoresis. The gel images were analyzed and the protein spots of differential expression were manually cut for protein annotation using mass spectrometry.

**Results:**

In total, 12 protein spots were excised and subjected to protein identification. Associated with KCS, six protein spots were a downregulated protein, namely, lysozyme. The other six protein spots were upregulated in KCS dogs, consisting of heat shock protein beta-1, protein S100-A12, and keratin type II cytoskeletal 1 and 5. After treatment with CsA for 45 days, the lysozyme protein was still decreasing and the inflammation protein (S100-A12) was not identified.

**Conclusion:**

Inflammatory tear proteins and proteins involved in cellular stress were present in KCS dogs and appeared to be reduced in medicated eyes. Treatment with topical CsA in the short term may not improve the activity of antibacterial proteins. Changes in the expression patterns of these four proteins might be useful for disease severity and progression assessment, as well as for exploring a novel method for dry eye management in dogs.

## Introduction

Research in the analysis of tears film composition, quality, and the relationship to the health of the ocular surface has been established for several years [1]. Keratoconjunctivitis sicca (KCS) known as “dry eye” has been estimated to affect between 5% and 50% of people worldwide [2]. This disease also occurs in a variety of dog breeds in Thailand. The breed prevalence of immunomediated KCS in dogs is American Cocker Spaniels, Bloodhounds, English Bulldogs, English Cocker Spaniels, English Springer Spaniels, Cavalier King Charles Spaniels, Boston Terriers, Lhasa Apsos, Miniature Schnauzers, Pekingese, Pugs, Samoyeds, Shih Tzus, and West Highland White Terriers [3]. KCS is a chronic inflammatory disease leading to a deficiency in the aqueous component of the lacrimal film, which promotes conjunctivitis, keratitis, and progressive corneal disease, including secondary corneal ulcers, resulting in the risk of vision loss [4,5]. To date, the diagnosis of quantitative KCS has been based on clinical signs and Schirmer’s tear test (STT). Clinical signs of KCS include conjunctivitis, keratitis, and ocular discharge. Normal reported STT values in dogs are 15-20 mm/min [6]. An STT result of <10 mm/min, in addition to clinical signs, is indicative of KCS. An STT result of <5 mm/min can be seen in severely affected dogs [4]. The most common cause of KCS in dogs is a probable autoimmune disorder of the lacrimal glands causing increased numbers of lymphocytes and plasma cells with acinar atrophy in the glands [3,7]. The first-line treatment for KCS in dogs has been topical ocular administration of cyclosporine A (CsA), an immunomodulatory agent that suppresses T-lymphocyte activation by reducing the production of the lymphokine interleukin-2 and formation of specific interleukin-2 receptors by helper T-lymphocytes resulting in reduced inflammation and increased tear production [8]. The study of dry eye syndrome in human found the primary proteins such as lysozyme, lactoferrin, and lipocalin was reduced but serum-derived proteins, such as serum albumin, IgG, and ceruloplasmin, were increased [9]. Many reports have mentioned the efficacy of topical CsA treatment to improve the STT in dogs with dry eye [10,11]; however, the alterations in the tear protein profile of the dog still remain unknown.

In humans, early efforts tried to use several techniques of tear protein analysis as an objective test for the diagnosis of dry eye and other ocular and systemic diseases [12,13]. Proteomics, the study of an entire set of proteins expressed by a genome, cell, tissue, or organism at a certain time, is used in tear protein study. For example, Grus *et a*l. [14] used surface-enhanced laser desorption ionization-mass spectrometry (SELDI-TOF-MS) to profile tear proteins from a human with dry eyes. In addition, they identified a potential tear biomarker which revealed an increase in inflammatory-related proteins and a decrease in proteins that may be protective. Human tear fluid is increasingly being used to detect protein biomarkers related to eye diseases such as dry eye [15], keratoconus [16], Graves ophthalmopathy [17], and diabetic retinopathy [18]. A tear protein profile was established in dogs by Winiarczyk *et al*. in [19]. There have been several applications of tear protein analysis including to develop potential cancer markers for diagnosis or management of canine cancers [20]. Proteomics may hold the key to unlocking biomarkers for various veterinary pathologies and diagnoses.

Although a recent study evaluated dog tear film proteome, no study has systematically investigated and characterized protein changes in KCS dogs and responsive proteins in KCS dogs treated with topical CsA. The current study initially examined tear films collected from both normal (control) and affected dogs using proteomic techniques, namely, two-dimensional electrophoresis (2-DE) and MS. The analysis of the differentially expressed tear proteins can provide a better understanding of the physiology of dog tear film and the pathology of KCS as well as further exploring its potential application for the treatment of KCS and other ocular surface disorders.

## Materials and Methods

### Ethical approval

This study was approved by the ethics committee of the Kasetsart University Research and Development Institute (ACKU59-VTN-005).

### Study period and location

The study was conducted from May 2018 to April 2019 in the Institute of Molecular Biosciences, Mahidol University and Faculty of Veterinary Technology, Kasetsart University.

### Study samples

Tear samples were collected from dogs of either gender and of any breed. The age of the dogs ranged from 2 to 13 years (mean 6.78 years). The vision of all dogs was examined by an obstacle test. Ophthalmic examination was performed, including menace response, dazzle reflex, and pupillary light reflex. The anterior segment of the eyes was examined using a portable slit lamp biomicroscope (SL-15, Kowa Optimed, Japan). Intraocular pressure was measured using a tonometer (TonoVet, iCare, Finland). The subjects were divided into three groups. The control group (G1) consisted of 15 dogs (two mixed breeds, three Shih Tzu, two Pomeranian, two Thai Ridgeback, and one each of Poodle, English Cocker Spaniel, Chihuahua, Labrador Retriever, French bulldog, and Jack Russell) classified as healthy based on an STT value more than 15 mm/min with no abnormal ocular signs or diseases. The KCS group (G2) consisted of 13 dogs (three mixed breeds, two French bulldogs, three Shih Tzu, two English Cocker Spaniel, and one each of Maltese, Poodle, and Pomeranian) diagnosed with KCS based on an STT value ≤10 mm/min. The G3 consisted of 10 of the 13 dogs from G2 that were first diagnosed as KCS and then received topical CsA (2% concentration in corn oil) twice daily for 45 days. The cyclosporine 2% was prepared by diluting the 100 mg/tab cyclosporine for oral administration (Atopica^®^, R.P. Scherer GmbH and Co.KG., Eberbach/Baden, Germany), using corn oil. The mixture was filtered through a 0.45 mm, Whatman^®^ membrane filter nylon. In severe cases (STT <5 mm/min), when considered necessary by the veterinarian, dogs were treated with commercial artificial tears 3 times a day. All dogs were given a general physical examination and general hematology and serum biochemistry. Only dogs with normal hematology and serum biochemistry values were included in this study. Dogs in the G2 group were excluded from the study if they had ever been treated previously with topical or systemic CsA or with any of the following drugs within 14 days before the study: Topical or systemic corticosteroids, atropine, antihistamines, pilocarpine, or sulfa-containing drugs, essential fatty acids or general anesthetics. Other exclusion criteria included the presence of any systemic disease other than dermatological disorders or of any ocular diseases affecting the ocular surface other than those related to orbital conformation in brachycephalic breeds. The study did not include dogs in which KCS had been determined to be congenital or secondary to neuroparalysis, secondary to surgery of the nictitans gland, due to distemper, or to the use of lacrimotoxic drugs.

Tear samples were collected from all groups using STT type I. The Schirmer strips were inserted for 5 min in the middle of the lower eyelid without anesthesia. Then, the strips were cut into small pieces and placed in elution buffer consisting of 200 mL of lysis buffer with protease inhibitors (8M urea, 2M thiourea, 4% CHAPS, 50 mM dithiothreitol, 0.1% Triton X-100, and 1X protease inhibitor cocktails) and stored at −20°C.

### Tear protein extraction

To be free of salt or other disturbing agents and provide an appropriate concentration for 2-DE analysis, tear proteins were extracted from STT strips and immediately precipitated. The eluted tear proteins were pooled from each group and centrifuged at 15,000 rpm at 4°C for 5 min. The supernatant was precipitated using the chloroform-methanol method. Briefly, 250 μL of protein solubilized in lysis buffer were incubated with 600 μL methanol, 150 μL chloroform, and 450 μL deionized water. The mixture was centrifuged at 14,000 rpm at 4°C for 5 min. The protein pellet was rinsed with 1 mL methanol and then air-dried, after which the pellets were rehydrated in lysis buffer. The eluted proteins were incubated in an ultrasonic bath for 30 min and centrifuged at 12,000 rpm at 4°C for 10 min. The supernatant was kept at −20^o^C until used. The tear protein concentration was determined using a Bradford assay at a wavelength of 280 nm.

### 2D gel electrophoresis

A sample of 120 mg of pooled protein from each group was separated in immobilized pH gradient strips (GE Healthcare, USA) 13 cm in length and with non-linear pH 3-10 range. The first-dimensional separation was carried out on an Ettan IPGphor II electrode platform (GE Healthcare) using an optimized focusing profiled of an increasing voltage to 20,000 V/h. Once the strips were focused, an equilibration step was performed using equilibration buffer (75 mM Tris-Cl, pH 8.8, 6 M urea, 2% SDS, 30% glycerol, and 0.002% bromophenol blue) containing 10 mg/mL DTT for 15 min followed by equilibration buffer containing 25 mg/mL iodoacetamide for 15 min with agitation at room temperature (26-36^o^C). The second-dimension SDS-polyacrylamide gel electrophoresis separation was performed using 12.5% gradient gel on a mini VE vertical electrophoresis system (GE Healthcare) with a constant voltage of 120 V for 120 min. The gels were stained with a solution containing 20% methanol, 10% ammonium sulfate, 2% phosphoric acid, and 0.001% colloidal Coomassie blue G250 with agitation for overnight. The gel images were captured on an ImageScanner II (GE Healthcare). The differential protein spots from triplicate gels of each group were analyzed using the Image Master 2-D Platinum software version 5.0 (GE Healthcare). The spots detected were statistically listed based on the percentage intensity volume based on the p-value for analysis of variances (p<0.05). Then, the protein spots showing statistically significant differences in protein expression were manually cut from the gels and subjected to gel digestion and MS.

### MS

The protein spots from 2-DE were destained using 50% acetonitrile in 50 mM ammonium bicarbonate. Protein reduction and alkylation were made using a final concentration of 4 mM dithiothreitol and 10 mM iodoacetamide, respectively. Individual gel pieces were subjected to tryptic digestion in a peptide mixture using an Ultimate 3000 nano-LC system (Dionex; Camberley, UK) equipped with an Acclaim PepMap RSLC (Thermo Scientific, Waltham, MA) for peptide separation. Subsequently, a micrOTOF-Q (Bruker Daltonics, Bremen, Germany) was coupled with the LC and the eluted peptides were analyzed online. The MASCOT search engine 2.2 (Matrix Science, Ltd. UK) was used for protein identification. The search parameters were set as the Swiss-Prot database, one miss cleavage, and trypsin digestion. Only proteins above the 95% confidence interval were reported in this research.

## Results

The mean (±SD) STT values in G1, G2, and G3 were 21.3±0.9, 4.0±0.4, and 16.1±0.6 mm/min, respectively. Statistically significant (p<0.001) differences in STT values were observed within the G1-G2 and G2-G3 groups. For the G3 groups, 10 of 13 dogs responding to CsA treatment were defined with STT values increases to more than 10 mm/min. Observed clinical signs of KCS dogs in the G2 group include corneal pigmentation, mucopurulent ocular discharge, corneal vascularization, and conjunctival hyperemia.

**Figure-1:**
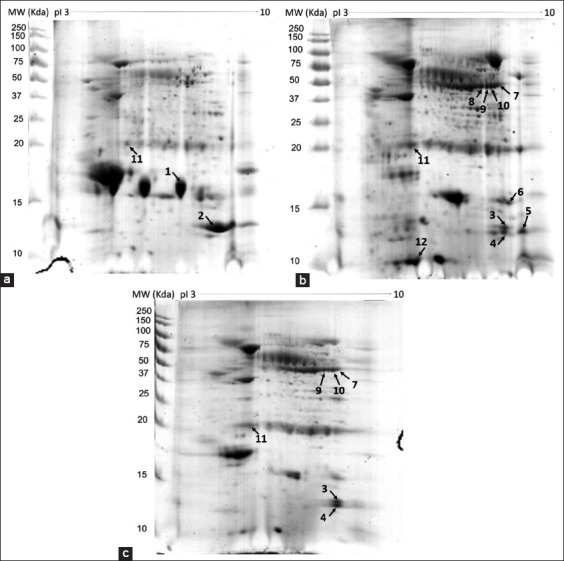
Protein expression profiles in dog tears. Two-dimensional electrophoresis analysis of 120 μg extracted proteins was resolved using isoelectric focusing at pH 3-10 non-linear with 7 cm IPG strip, followed by 12.5% SDS-PAGE compared with pooled tear protein from healthy dogs (a), KCS dogs (b), and dogs with CsA treatment (c). The Precision Plus Protein™ Unstained Standards (Bio-Rad) were used as a protein marker. The arrows and numbers indicate the altered protein expression.

To investigate differences in the protein composition of tears among the different dog groups, extracted tear samples were analyzed using two-dimensional gel electrophoresis. The intensities of the protein spots were calculated. Representative gels for the pools of normal dog tears, tears from KCS dogs, and tears from CsA-treated dogs are shown in Figure-1. Tear samples from all groups had a similar distribution of major tear proteins in the 2-DE maps, but some proteins appeared to alter in concentration. Several protein spots in the 2-DE analysis showed significant differences in protein patterns (Spot nos. 1-12). Three spots from healthy dogs (spot nos. 1, 2, and 11), 10 spots from KCS dogs (spot nos. 3-12), and six spots from CsA dogs (spot no. 3, 4, 7, and 9-11) were selected and excised for protein identification using MS.

The identified proteins are shown in Table-1. Spots 1-6 were identified as the lysozyme C protein. Based on the intensity of staining (using Coomassie blue), the amount of lysozyme C in KCS dogs appeared to be less than in the healthy dog tears. Many of the protein groups displayed charge heterogeneity, most likely due to post-translational processing [16]. Spot nos. 7, 8, 9, and 10 in KCS dog tears and spot nos. 7, 9, and 10 in CsA dogs were identified as keratin protein which was not detected in healthy dogs in the same area. Heat shock protein beta-1 (HSPB1) (spot no. 11) was detected in all groups of dog tears but had a higher intensity of staining in KCS dog tears than in the healthy and CsA dog tears. Another major tear protein, protein S100-A12 (spot no. 12), detected in the low-molecular-weight area was found only in KCS dog tears. Interestingly, protein identification in tears from dogs that had received topical CsA twice daily for 45 days (G3 dog group) had similar tears to the KCS dogs, except for the significant decrease of lysozyme C and S100-A12.

**Table-1:** Identification of significantly different expressed proteins in dog tears. Mass spectrometry analysis of selected protein spots of healthy dogs (G1), KCS dogs (G2), and CsA-treated dogs (G3) was obtained from 2-D separation. Changes in tear proteins are classified as upregulated and downregulated.

Spot no.	Identified proteins	Biological function	Protein score	Mass (kDa)	pI	KCS dogs	CsA-treated dogs	p-value
1	Lysozyme C	Antimicrobial	479	14.5	8.6	Downregulated	Downregulated	0.004
2	Lysozyme C	Antimicrobial	2294	14.5	8.6	Downregulated	Downregulated	0.004
3	Lysozyme C	Antimicrobial	1048	14.5	8.6	Downregulated	Downregulated	0.009
4	Lysozyme C	Antimicrobial	1072	14.5	8.6	Downregulated	Downregulated	0.005
5	Lysozyme C	Antimicrobial	642	14.6	9.1	Downregulated	Downregulated	0.001
6	Lysozyme C	Antimicrobial	642	14.6	9.1	Downregulated	Downregulated	0.021
7	Keratin, type II cytoskeletal 1	Establishment of skin barrier	50	65.9	8.2	Upregulated	Upregulated	0.009
8	Keratin, type II cytoskeletal 5	Keratinization	72	61.7	7.6	Upregulated	Upregulated	0.013
9	Keratin, type II cytoskeletal 1	Establishment of skin barrier	836	65.9	8.2	Upregulated	Upregulated	0.020
10	Keratin, type II cytoskeletal 1	Establishment of skin barrier	50	65.9	8.1	Upregulated	Upregulated	0.009
11	Heat shock protein beta-1	Response to cellular stress	133	22.9	6.2	Upregulated	Downregulated	0.038
12	Protein S100-A12	Inflammatory response	36	10.7	5.7	Upregulated	Downregulated	0.047

## Discussion

This was the first study reporting on comparative proteome profiles of tears from normal dogs, dry eye dogs, and dogs on dry eye medication. The STT values (21.3±0.9 mm/min) in normal groups of dogs measured from a variety of dog breeds related to normal ranges of STT in brachycephalic (20.1±3.4 mm/min) and non-brachycephalic dogs (23.3±5.7 mm/min) [21]. However, there was no comparative study on specific tear proteins in the different cephalic conformations in dogs. The tear proteins play an important role in the maintenance of the ocular surface. Proteins in tears are dynamic and sensitive to many factors such as the collection method, proteomic analytical techniques, and physiological states [22,23]. For the physiological states, the tear film proteins were previously analyzed in dogs with the peculiar shape of the skull such as pugs which reported a high number of eye diseases, known as a brachycephalic ocular syndrome. Protein abundant in serum increased with severity of ocular lesions in 2-DE protein patterns of single patterns [24]. In 2015, a canine tear proteome was explored using MS which identified 125 proteins in the tear film samples from six healthy dogs [19]. In that report, one-dimensional sodium dodecyl sulfate-polyacrylamide gel electrophoresis (1D SDS-PAGE) was used as the first step to separate the tear proteins. Then, each visualized band was analyzed using matrix-assisted laser desorption/ionization time-of-flight tandem MS (MALDI-TOF-MS/MS) to identify the tear protein. In the present study, two-dimensional gel electrophoresis was used to identify the altered tear proteins. Because of the different techniques, there was variation in the tear proteins identified.

Protein spots of lysozymes were detected variably in the 2-DE map of tear fluid samples from all dog groups. Lysozyme has important roles in tear fluids to protect the eye from bacterial infection. It is manufactured in the acinar cells of the lacrimal glands. Lysozyme has an antibacterial action with hydrolyzing glycosidic bonds, particularly those of certain Gram-positive bacteria, in the bacterial cell walls [25]. Lysozyme occurs as an abundant protein in all tear fluids of humans, cows, sheep, and camels [26]. A previous study found that the lysozyme concentrations in tears varied depending on the animal species and may contribute to differing susceptibilities to ocular disorders [27]. The current study identified a statistically significant decrease in lysozyme in the KCS dogs, indicating not only a decrease in lacrimal gland synthesis but also reduced lysozyme could lead to an increased predisposition to the development of infection in the ocular surface. Similarly, a human study reported the amount of lysozyme in human tears also decreased in early dry eye [14]. The tear lysozyme was used as one of the potential biomarkers for diagnosis of human dry eye and mucosal immune competence [14,23,28,29]. In the current study, we collected tear samples of the G3 dogs from the KCS dogs who had received CsA for only 45 days, at which time, the concentration of lysozyme was still decreasing. This indicated that it might need longer than 45 days for CsA dogs to improve the amount of tear lysozyme they produce.

HSPB1 or HSP27 is one of the smallest heat shock proteins which was originally identified as a stress-responsive protein required to deal with thermal and other proteotoxic stresses. Many members of HSPB1 show different expressions between the tear sample types identified proteins [20] and high expression in skeletal and cardiac muscle and are also found in many other tissues [30]. HSPB1 is one of the tear proteins identified in people with diabetic retinopathy and can serve as a future early diagnostic tool for this disease [31]. Moreover, HSPB1 expression increased in glaucoma patients, which involved the progressive loss of retinal ganglion cells and optic nerve degeneration [32]. In the current study, this protein was found in all groups of tear samples, with significantly increased expression in dogs with KCS, indicating cellular stress, while the expression decreased in KCS dogs after treatment with topical CsA for 45 days.

Our 2-DE results revealed increased levels of the inflammation-associated protein, S100-A12, in the KCS group. This protein has been reported in normal cow tears [26]. S100-A12 is the major pro-inflammatory and stress-related proteins of the S100 family, including S100-A8 and S100-A9. The protein directly activates endothelial cells, mononuclear phagocytes, and lymphocytes through interaction with the receptor for advanced glycation and products [33]. Elevated levels of S100-A12 have been reported in humans with autoinflammation, during local and systemic inflammation [34] and in dogs with canine mammary tumors [35]. Interestingly, the S100-A12 protein was downregulated following CsA treatment, which agreed with a previous study that reported decreasing clinical signs of conjunctival and corneal inflammation after CsA treatment [11]. Moreover, post-CsA treatment, there was no intensity of protein spots in the area of the S100-A12 protein, which is similar to the protein pattern in the protein tears of healthy dogs. Our findings suggested that the protein associated with the inflammation, S100-A12, might be a candidate biomarker that could be useful for determining KCS severity and treatment effectiveness.

This study found the expression of the keratin type II cytoskeletal 1 and 5 proteins in tear samples of KCS and CsA-treated dog. These proteins were observed in both human and dog tears [19]. In humans, the keratin type II cytoskeletal 1 was downregulated in the tear samples of patients with keratoconus, a progressive thinning of the cornea giving rise to a cone-shaped cornea, instead of the normal elliptical shape [36] but this protein was not significantly upregulated in the disease status of Sjögern patients with dry eye syndrome [37,38]. The keratin type II cytoskeletal 5 protein was also present in the tears of people with keratoconus [39]. In humans, the keratin proteins are normally found in the epidermal layer of skin and are not necessarily present in the tear film; therefore, a possible presence in tears could be caused by the habit of eye rubbing in people experiencing eye discomfort [39]. This was the first report of an increase of keratin protein in KCS dog tears. Further study could investigate how this proteomic tear change is involved in the pathogenic mechanism in KCS dogs.

In this study, we selected tear protein spots which produced different staining from the three conditions of dogs tested protein identification. There is a possibility that unidentified proteins have possibly been missed, especially less abundant tear proteins.

## Conclusion

Proteomic analysis of dog tears can provide insight into changes in the tears in KCS dogs. Our findings revealed that the composition of tear proteins in dry eye dogs was different from that of healthy dogs and also that treatment of dry eye using topical CsA for 45 days might not improve the tear proteins to normal levels. The changes in the expression patterns of proteins may be helpful in the diagnosis of dry eye disease and monitoring their response to therapeutic intervention. However, these proteins need to be objectively measured and validated for their sensitivities and specificities in the future study.

## Authors’ Contributions

MS, RR, and AT: Designed the study, revised and finalized the manuscript for submission. MS and PH: Collected samples and did 2-DE gel electrophoresis. OR: Analyzed the mass spectrometry. MS and RR: Drafted and revised the manuscript. All authors read and approved the final manuscript.
